# Supplementary material for: Effects of Dietary Tannic Acid on Obesity and Gut Microbiota in C57BL/6J Mice Fed with High-Fat Diet
Source: Foods. 2022 Oct 23;11(21):3325. doi: 10.3390/foods11213325 (PMC9659306; doi:10.3390/foods11213325)
Supplement: Supplementary file 1 [file foods-11-03325-s001.zip › foods-1946277-supplementary.pdf]

Table S1. Target gene primer sequence.

Target Gene	Annealing Temperature	Primer Sequence（5′ － 3′ ）
PPAR-γ	56℃	F:CTTTACCACGGTTGATTTCTC
		R:CAGGCTCTACTTTGATCGC
PPAR-α	60℃	F:GTACGGTGTGTATGAAGCCATCTT
		R:GCCGTACGCGATCAGCAT
SREBP	60℃	F:TCGGCACCCGCTGCTTTA
		R:GGGTCCCTGAGAAGCCTGAAG
LPL	60℃	F:CCACAGCAGCAAGACCTTC
		R:AGGGCGGCCACAAGTTTG
FAS	60℃	F:TGCTCCCAGCTGCAGGC
		R:GCCCGGTAGCTCTGGGTGTA
ACC	60℃	F:TGGCTGGCTGGACAGACTGATAG
		R:CGCTATTCCGCAGGCTCACATC
AMPK	60℃	F:GGTGTTATCCTGTATGCCCTTCT
		R:TGTCTTTGATAGTTGCTCGCTTC
CPT-1	60℃	F:GTGACTGGTGGGAGGAATAC
		R:GAGCATCTCCATGGCGTAG
GADPH	60℃	F:TGGCCTTCCGTGTTCCTAC
		R:GAGTTGCTGTTGAAGTCGCA
β-actin	60℃	F:ACTGCCGCATCCTCTTCCTC
		R:AAAGAGCCTCAGGGCATCGG
IL-6	60℃	F: CTGCAAGAGACTTCCATCCAG
		R:AGTGGTATAGACAGGTCTGTTGG
TNF-α	60℃	F: TGGCCCAGACCCTCACACTCAG
		R: ACCCATCGGCTGGCACCACT
ZO-1	58℃	F: CTTCTCTTGCTGGCCCTAAAC
		R: TGGCTTCACTTGAGGTTTCTG
Occludin	60℃	F: CACACTTGCTTGGGACAGAG
		R: TAGCCATAGCCTCCATAGCC
